# Influence of cellular models and individual factor in the biological response to head CT scan exams

**DOI:** 10.1186/s41747-022-00269-x

**Published:** 2022-04-07

**Authors:** Clément Devic, Larry Bodgi, Laurène Sonzogni, Frank Pilleul, Hervé Ribot, Charlotte De Charry, François Le Moigne, Didier Paul, Fanny Carbillet, Mélodie Munier, Nicolas Foray

**Affiliations:** 1 Institut National de la Santé et de la Recherche Médicale, U1296 Radiations Defense, Health and Environment Centre Léon-Bérard, 69008 Lyon, France; 2 FibermetrixTM SAS, 7 Allée de l’Europe, 67960 Entzheim, France; 3 Radiation Oncology Department, American University of Beirut Medical Center, Beirut, 1107 2020 Lebanon; 4 Service de Radiologie, Centre Léon Bérard, 28 rue Laennec, 69008 Lyon, France; 5 Service de Radiologie, Hôpital d’Instruction des Armées, Desgenettes », Boulevard Pinel, 69003 Lyon, France; 6 ALARA Expertise SAS, 7 Allée de l’Europe, 67960 Entzheim, France

**Keywords:** DNA breaks (double-stranded), Li-Fraumeni syndrome, Neurofibromatosis 1, Radiobiology, Tomography (x-ray computed)

## Abstract

**Background:**

While computed tomography (CT) exams are the major cause of medical exposure to ionising radiation, the radiation-induced risks must be documented. We investigated the impact of the cellular models and individual factor on the deoxyribonucleic acid double-strand breaks (DSB) recognition and repair in human skin fibroblasts and brain astrocytes exposed to current head CT scan conditions.

**Method:**

Nine human primary fibroblasts and four human astrocyte cell lines with different levels of radiosensitivity/susceptibility were exposed to a standard head CT scan exam using adapted phantoms. Cells were exposed to a single-helical (37.4 mGy) and double-helical (37.4 mGy + 5 min + 37.4 mGy) examination. DSB signalling and repair was assessed through anti-γH2AX and anti-pATM immunofluorescence.

**Results:**

Head CT scan induced a significant number of γH2AX and pATM foci. The kinetics of both biomarkers were found strongly dependent on the individual factor. Particularly, in cells from radiosensitive/susceptible patients, DSB may be significantly less recognised and/or repaired, whatever the CT scan exposure conditions. Similar conclusions were reached with astrocytes.

**Conclusions:**

Our results highlight the importance of both individual and tissue factors in the recognition and repair of DSB after current head CT scan exams. Further investigations are needed to better define the radiosensitivity/susceptibility of individual humans.

**Supplementary Information:**

The online version contains supplementary material available at 10.1186/s41747-022-00269-x.

## Key points

- Head computed tomography (CT) scan exposure discriminates individuals with deoxyribonucleic acid double-strand breaks (DSB) as endpoints.
- Cells from radiosensitive/susceptible patients elicit more DSB after head CT scan exposure.
- The justification of CT scans should take into account individual factors.

## Background

To date, computed tomography (CT) scan exams, represents the largest cause of medical exposure to ionising radiation (IR) [1, 2]. For the last decade, the average annual effective dose received in a medical context has increased continuously [1, 3]. These statements raise questions about the justification of the medical exposure to IR and the risks potentially due to CT scan exams [4–6]. More recently, a decree of the French National Nuclear Safety Authority encourages radiologists to reduce or better justify the diagnosis involving IR by taking into account individual risk factors of the patients to be imaged [7].

Numerous radiobiological studies have been performed to better understand and evaluate the biological consequences of CT scan exposures [8–10]: Since chromosome and deoxyribonucleic acid (DNA) damage are likely involved in the response to IR, the great majority of the research groups have investigated the chromosome and DNA damage induced by CT scan exposure in *in vitro* or *ex vivo* lymphocytes [8–10]. However, the radiation-induced (RI) cancers are not limited to leukaemia and lymphocytes are not necessarily the most appropriate cellular models to evaluate the RI risks linked to CT scan exposure [4, 5]. Since brain tumours are often cited as cancers potentially induced by CT scan exams [4, 11, 12], brain astrocytes should also be used in the radiobiological characterisation of CT scan exposure. Similarly, fibroblasts, that represent the majority of cells in human body, may be also a useful cellular model. However, no radiobiological data has been obtained from astrocytes and fibroblasts exposed to current CT scan exposure conditions.

There is increasing evidence that individual factors, notably mutations of genome maintenance genes, may significantly influence the follow-up of exposed patients and contribute to increase the RI risks linked to CT scan exposure. Again, few studies, if any, have raised the question of the relative contribution of the individual factor in the biological response to CT scan exposures [13–15].

**Fig. 1:**
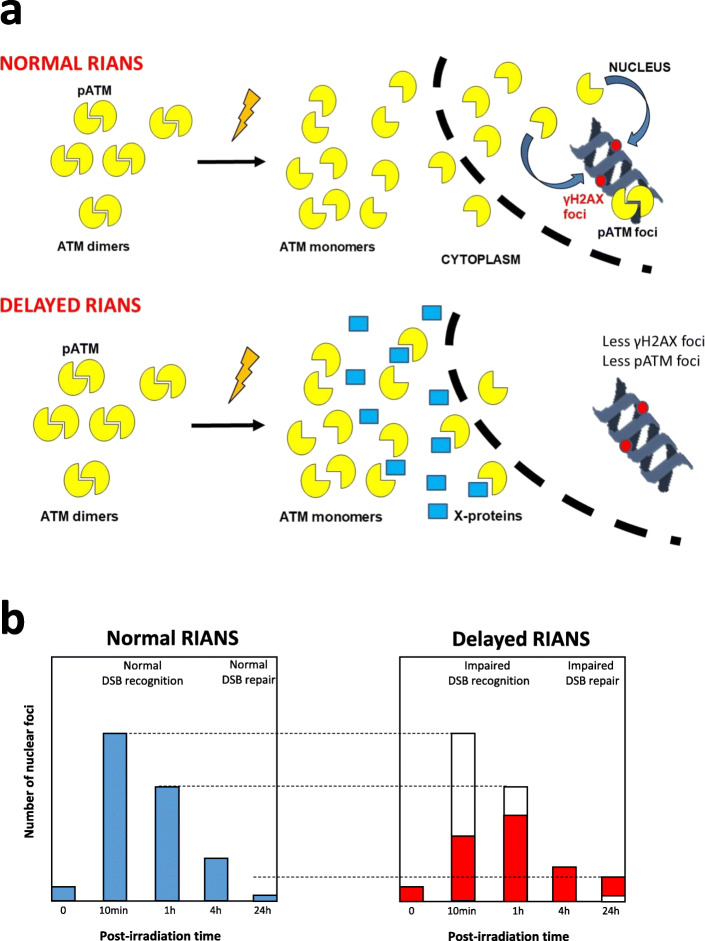
The radiation-induced ATM nucleoshuttling: RIANS model and its current biomarkers. **a** In the frame of the RIANS model, IR induce the monomerisation of the ATM dimers in cytoplasm. The resulting ATM monomers diffuse in the nucleus and phosphorylate the H2AX histone variant molecular (γH2AX) at DSB sites, which triggers the formation of nuclear γH2AX foci, easily quantifiable by immunofluorescence. This is the recognition step. The recognised DSB are repaired by the non-homologous end-joining, the major DSB repair pathway in humans (see refs. [8, 16–21]). During the DSB repair process, two ATM monomers reassociate on the DSB sites and form the nuclear autophophorylated ATM (pATM) foci, also visible by immunofluorescence. **b** In the kinetics of γH2AX or pATM foci appearance, the early (10 min, 1 h) post-irradiation times provide information on the functionality of the DSB recognition step while the late (24 h) ones provide information on the functionality of the DSB repair step. *ATM* Ataxia telangiectasia mutated gene/protein, *DSB* Deoxyribonucleic acid double-strand breaks, *RIANS* Radiation-induced nucleoshuttling of the ATM protein, *γH2AX* Phosphorylated forms of the H2AX histone variant molecular

A unified model of the individual response to IR, relevant for both high and low doses, and based on the RI nucleoshuttling of the ataxia-telangiectasia mutated (ATM) protein kinase (RIANS), was proposed recently [16–18] (Fig. 1a). Any delay in the RIANS may lead to radiosensitivity (RI adverse tissue reactions proneness), radiosusceptibility (RI cancer proneness), and/or radiodegeneration (RI aging proneness) [15, 17]. By using immunofluorescence technique, in the frame of the RIANS model, the nuclear foci formed in irradiated cells by the phosphorylated forms of the H2AX variant histone (γH2AX) and ATM (pATM) proteins at the DSB sites may serve as useful endpoints to characterise any specific exposure to IR. Particularly, the γH2AX and pATM foci observed early (10 min, 1 h) after irradiation provide information about the DSB recognition process while the late (24 h) ones characterise the DSB repair step (Fig. 1b) [17, 22].

This study aims to assess the DSB recognition and repair induced by current head CT scan exposure conditions in nine untransformed skin fibroblasts showing a wide spectrum of radio-sensitivity/susceptibility and four untransformed brain astrocytes providing from the same donor, by using γH2AX and pATM foci as endpoints. Physical dosimetry was insured by a new generation optical scintillating fibre dosimeter [23].

## Methods

### Cells

Human untransformed fibroblasts were cultured as monolayers in the conditions detailed elsewhere [16]. The fibroblasts were exposed at passages lower than 15. All the experiments were performed with cells in plateau phase of growth (95–99% in G0/G1) to overcome any cell cycle effect. Some of the fibroblast cell lines used in this study were provided from a collection of cells derived from radiosensitive and/or radiosusceptible patients, the COPERNIC collection [16]. This collection was approved by the regional ethical committee in respect of the national regulatory procedures. Cell lines were declared under the agreement numbers DC2008-585, DC2011-1437, and DC2021-3957 to the Ministry of Research. The COPERNIC database that gathers radiobiological data of these cell lines was protected under the reference IDDN.FR.001.510017.000.D.P.2014.000.10300. All the anonymous donors were informed and gave signed and consent according to the ethics recommendations [16].

Among the COPERNIC cell lines, the 200CLB cell line derived from an apparently healthy patient and served as radioresistant control. The 01HNG, 02HNA and 13HNG cell lines derived from patients who showed severe tissue reactions after radiotherapy [16] and served as representative radiosensitive examples. The RACKHAM01, RACKHAM12, and RACKHAM39 cell lines were derived from three different neurofibromatosis type 1 (*NF1*^*+/−*^ mutated patients). The 85MA cell line derived from a Li-Fraumeni syndrome (*p53*^*+/−*^ mutated) patient was a kind gift from D. Scott (Manchester, UK). The GM03399 cell line was derived from a heterozygous ataxia telangiectasia (*ATM*^*+/−*^ mutated) patient and was purchased from Coriell Institute (Camden, NJ, USA). These last five cell lines served as representative radiosusceptible (high cancer risk) examples. The origin and the major clinical features of the nine fibroblast cell lines have been gathered in the Table 1.

**Table 1:** Major clinical features of the cell lines used in this study

Cell lines	Cell type	Known genes mutation	Cancer proneness	Radiobiological status
200CLB	Fibroblast	Apparently healthy	ND	Radioresistance
RACKHAM01	Fibroblast	Neurofibromatosis type 1^+/−^	Central and peripheral nervous system tumours	Radiosensitivity and radiosusceptibility
RACKHAM12	Fibroblast	Neurofibromatosis type 1^+/−^	Central and peripheral nervous system tumours	Radiosensitivity and radiosusceptibility
RACKHAM39	Fibroblast	Neurofibromatosis type 1^+/−^	Central and peripheral nervous system tumours	Radiosensitivity and radiosusceptibility
01HNG	Fibroblast	ND (cancer patient)	ND	Radiosensitivity
02HNA	Fibroblast	ND (cancer patient)	ND	Radiosensitivity
13HNG	Fibroblast	ND (cancer patient)	ND	Radiosensitivity
GM03399	Fibroblast	Ataxia-telangiectasia mutated^+/−^	Mainly leukaemia, lymphoma	Radioresistance and radiosusceptibility
85MA	Fibroblast	p53^+/−^	Breast, brain, leukaemia, sarcoma	Radioresistance and radiosusceptibility
HA	Astrocyte	Apparently healthy	ND	ND
HA-sp	Astrocyte	Apparently healthy	ND	ND
HA-h	Astrocyte	Apparently healthy	ND	ND
HA-bs	Astrocyte	Apparently healthy	ND	ND

*ND* Not determined

Four brain cell lines, HA, HA-sp, HA-h, and HA-bs, providing from the same donor, were used in this study. These human untransformed astrocytes were purchased from the Sciencell Research laboratories (Carlsbad, CA, USA). HA, HA-sp, HA-h, and HA-bs were isolated from the cerebral cortex, spinal cord, hippocampus, and brain stems, respectively. Astrocytes were cultured in the conditions recommended by the manufacturer *i.e.*, in medium AM (#1801, Sciencell), supplemented with 20% fetal bovine serum (#0010; Sciencell) and penicillin/streptomycin solution (#0503; Sciencell). Astrocytes were exposed at passages lower than 10. All the experiments were performed with cells in plateau phase of growth (95–99% in G0/G1) to overcome any cell cycle effect. The origin and the major clinical features of the four astrocyte cell lines have been gathered in Table 1.

### Head CT scan exposure conditions

**Fig. 2:**
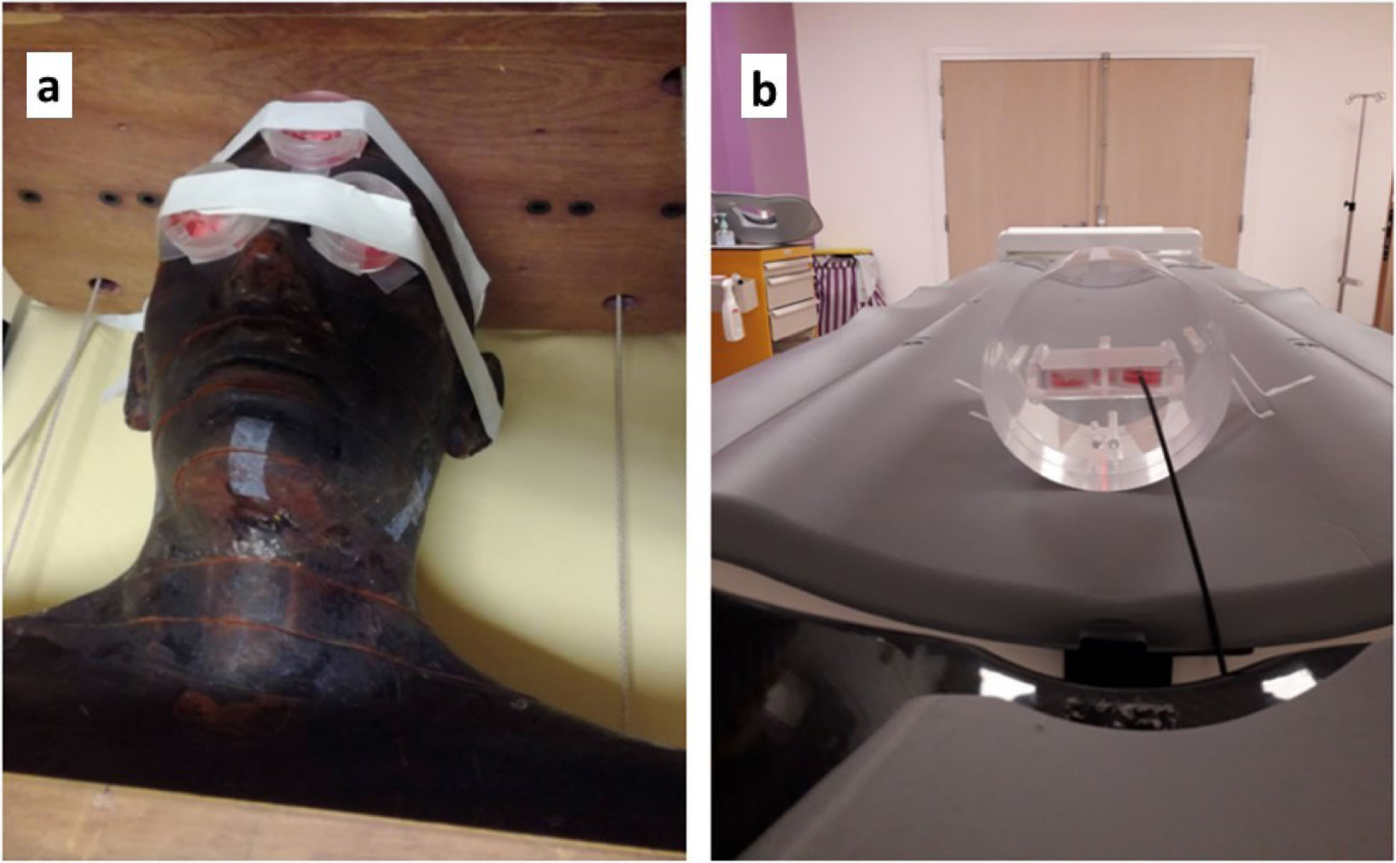
Representative images of the irradiation setup. **a** Anthropomorphic phantom used for fibroblasts irradiation. **b** PMMA phantom (longer width, 16 cm) used for astrocytes irradiation**.** In order to be clinically relevant, fibroblasts were exposed on the surface of the anthropomorphic head phantom and astrocytes were exposed inside the PMMA phantom. *PMMA* Poly(methyl methacrylate)

*In vitro* set up was characterised by a tissue-specific phantom. Fibroblasts were exposed on the surface of the anthropomorphic head phantom and astrocytes were exposed inside the poly(methyl methacrylate) 16-cm diameter phantom, both in Petri dishes 35 × 10 mm (#353001, Falcon, Deutscher, Bernolsheim, France) (Fig. 1). The dose was measured with a dosimeter based on scintillating fibre developed by the Fibermetrix company [23–25] (Fig. 2). Spiral CT scan was performed by using a Siemens Definition Edge apparatus (Siemens Healthineers, Erlangen, Germany) operated at 166/230 mAs, 120 kV, with a 1-s rotation time, a 0.6 pitch and a 3-mm collimation in Centre Léon-Bérard (Lyon, France). CT exposures were delivered with single-helical (37.4 mGy) or double-helical helical (37.4 mGy + 5 min + 37.4 mGy) to illustrate repeated helical series that can occur during a CT exam. Additional data were also obtained at Army Hospital Desgenettes (Lyon, France) (see “Results” section).

An intercomparison study with different CT scan machines was initiated. As a first step, the head CT scan exposure conditions described in Methods were applied to a Philips Brillance iCT 256 at the Army Hospital Desgenettes (Lyon, France). By comparing data from the Siemens Definition Edge CT scan machine at Centre Léon-Bérard (Lyon, France), the doses assessed at the surface of the phantom were not found different (34.6 ± 2.4 mGy and 37.4 ± 1 mGy and, respectively (*p* > 0.550), suggesting a low impact of the CT scan machines when comparing these two irradiators.

### Immunofluorescence analysis

The γH2AX and pATM immunofluorescence protocol for assessing DSB recognition and repair was described elsewhere [26–28]. Briefly, cells were fixed in paraformaldehyde for 15 min at room temperature and permeabilised in detergent solution for 3 min. Primary antibody incubations were performed for 1 h at 37 °C. Anti-*γH2AX*^*ser139*^ antibody (#05-636 Merck, Molsheim, France) was used at 1:800, the monoclonal anti-mouse anti-*pATM*^*ser1981*^ (#05-740 Merck) was used at 1:100. Incubations with anti-mouse fluorescein secondary antibodies provided by Sigma-Aldrich (L’Isle d’Abeau Chesnes, France) were performed at 1:100 at 37 °C for 20 min. Slides were mounted in 4′,6′ Diamidino-2-phenyl-indole-stained Vectashield (Vector Laboratories, Burlingame, CA, USA) and cells were counted using an X100 objective with a fluorescence BX51 Olympus microscope (Olympus-France, Rungis, France). For each of the three independent experiments, 100 nuclei were analysed. The patented procedures of foci scoring have been detailed elsewhere [28].

### Data processing and statistics

The data and statistical analyses were processed using MATLAB R2019a (MathWorks, Natick, MA, USA). Since each experiment is the result of 3 independent replicates with 100 nuclei scored, the general mean of the 3 means of each replicate was given with the standard error of the mean (SEM). By contrast, significance tests were done by grouping the 300 nuclei data for each cell line and condition. To compare two conditions with each other, a non-parametric Mann-Whitney-Wilcoxon test was used [29, 30]. For the γH2AX data, the number of foci in each cell line without irradiation, 10 min and 1 h post-irradiation was compared with the corresponding conditions of 200CLB for fibroblasts, and HA-h for astrocytes. The residual number of γH2AX foci was compared with the non-irradiated conditions for each cell line. For pATM data, the number of foci in each cell lines without irradiation and 10 min post-irradiation was compared with the corresponding conditions of 200CLB for fibroblasts, and HA-h for astrocytes [29, 30]. Kruskal-Wallis test was performed to compare the corresponding data of all fibroblasts together on the one hand, and all astrocytes together on the other hand, for both single- and double-helical irradiations [31]. For each test, the differences were considered statistically significant when the *p* value was lower than 0.050. Table S1 recapitulates all the significant differences observed.

## Results

### Radiobiological effects of single- and double-helical head CT scans on cutaneous fibroblasts

The average volumetric CT dose index (CTDIvol) was 29.6 ± 1.2 mGy. The average dose-length product (DLP) was 467 ± 17 mGy.cm. The average absorbed dose at the surface of the phantom was 37.4 ± 1 mGy and 75.1 ± 2.3 mGy for the single- and double-helical conditions, respectively.

**Fig. 3:**
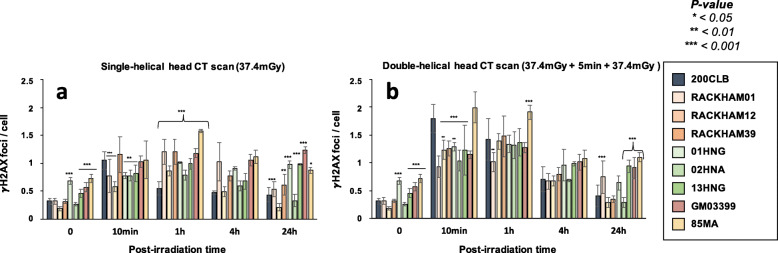
γH2AX foci in fibroblasts after single- and double-helical CT scan exposure. **a** Kinetics of γH2AX foci in fibroblasts after a single helical head CT scan, or (**b**) a double-helical head CT scan at the indicated times after exposure (t0 = non irradiated). Each data represents the mean ± SEM of 3 independent experiments. The asterisks shown at the non-irradiated conditions and at 10 min and 1 h post-irradiation times correspond to a statistically significant difference with the radioresistant control 200CLB (*p* < 0.05). The asterisks shown at 24 h post-irradiation correspond to a statistically significant difference with the non-irradiated conditions for the same cell lines. *CT* Computed tomography, *γH2AX* Phosphorylated forms of the H2AX histone variant, *SEM* Standard error of the mean

Without irradiation, the radioresistant 200CLB control fibroblasts showed 0.31 ± 0.05 spontaneous γH2AX foci per cell. Among the other tested fibroblasts, 4 cell lines (GM03399, 85MA, 01HNG, 13HNG) showed significantly more spontaneous γH2AX foci (*p* < 0.001), suggesting a higher genomic instability, although the number of γH2AX foci never exceeded 1 foci per cell. For the other cell lines, the numbers of spontaneous γH2AX foci were not found different from that of controls (Fig. 3a).

Ten minutes after a single-helical CT scan session, the average number γH2AX foci was found to be 1.05 ± 0.15 γH2AX foci per cell in the radioresistant controls. This value was not statistically different from the currently reported rate of DSB induced per Gy per human diploid fibroblast (37 ± 4 γH2AX foci per Gy per cell [27]: the expected value would have been 1.38 ± 0.15 γH2AX foci per cell after 37.4 mGy) (Fig. 3a). Five cell lines (RACKHAM01, RACKHAM12, 01HNG, 02HNA, and 13HNG showed a number of γH2AX foci significantly lower than radioresistant controls (*p* < 0.001, *p* < 0.001, *p* = 0.015, *p* = 0.005 and *p* = 0.020, respectively (Fig. 3a). In the other cell lines, the early DSB recognition was found normal for the doses applied.

In the radioresistant controls, the number of γH2AX foci reached its maximal value at 10 min post-irradiation, reflecting a normal DSB recognition, decreased thereafter, and reached a number of residual γH2AX foci at 24 h post-irradiation not different from that assessed before irradiation. The γH2AX foci kinetics of all the other cell lines differed from those of controls with a number of γH2AX foci reaching its maximal value at 1 h post-irradiation, suggesting a delay in the DSB recognition process and decreasing thereafter (Figs. 3a and S1a). At 24 h post-irradiation, two situations were encountered with the radio-sensitive/susceptible cell lines: (1) a subset of fibroblasts showed a slower rate of γH2AX foci disappearance and the number of γH2AX foci was found significantly higher than non-irradiated control, suggesting an impaired DSB repair process. This is the case of RACKHAM01, RACKHAM39, GM03399, 85MA, 01HNG, and 13HNG cell lines (Fig. 3a; *p* < 0.050); (2) another subset of fibroblasts did not show any statistically significant difference when comparing residual γH2AX foci with spontaneous ones, suggesting a normal DSB repair process. This is the case of RACKHAM12 and 02HNA (Figs. 3a and S1a).

When fibroblasts were irradiated in the double-helical head CT scan conditions, the number of γH2AX foci assessed 10 min post-irradiation increased significantly in a cell line-dependent manner (Figs. 3b and S1b; *p* < 0.001). In the radioresistant controls, there was two times more γH2AX foci after a double-helical than after single-helical CT scan, suggesting an additive dose-effect. By contrast, the γH2AX data ratio between double-helical and single-helical CT scan conditions was lower than 2 for RACKHAM01, RACKHAM39, 02HNA and GM03399 cells, suggesting that the lack of DSB recognition is so severe that the second helical CT scan view did not help in providing more ATM monomers in the nucleus of these cells to better recognise DSB. Conversely, in 01HNG, 85MA and RACKHAM12, the ratio between double-helical and single-helical CT scan conditions was found much higher, suggesting that the double-helical CT scan conditions may enhance the recognition of the RI DSB or trigger a supplementary induction of DSB (like the hyper-recombination phenomenon).

At 24 h after the double-helical CT scan exposure, two situations were encountered: (1) a subset of fibroblasts showed a number of γH2AX foci significantly higher than non-irradiated control, suggesting an impaired DSB repair process. This is the case of RACKHAM01, GM03399, 85MA, and 13HNG cell lines (Fig. 3b; *p* = 0.045); (2) another subset of fibroblasts did not show any statistically significant difference when comparing residual γH2AX foci with spontaneous ones, suggesting a normal DSB repair process. This is the case of the other cell lines (Fig. 3b). Table 2 recapitulates these findings.

**Table 2:** Major clinical features of the cell lines used in this study

Cell lines	Status	Single-helical scan		Double-helical scan	
		DSB recognition	DSB repair	DSB recognition	DSB repair
*Fibroblasts*					
200CLB	Apparently healthy	+	+	+	+
RACKHAM01	Neurofibromatosis type 1^+/−^	−	−	−	−
RACKHAM12	Neurofibromatosis type 1^+/−^	−	+	+	+
RACKHAM39	Neurofibromatosis type 1^+/−^	−	−	−	+
01HNG	Cancer patient	−	−	+	+
02HNA	Cancer patient	−	+	−	+
13HNG	Cancer patient	−	−	+	−
GM03399	Ataxia-telangiectasia mutated^+/−^	−	−	−	−
85MA	p53^+/−^	−	−	+	−
*Astrocytes*					
HA	Apparently healthy	−	−	−	−
HA-sp	Apparently healthy	−	+	−	−
HA-h	Apparently healthy	+	+	−	+
HA-bs	Apparently healthy	−	−	−	+

**Fig. 4:**
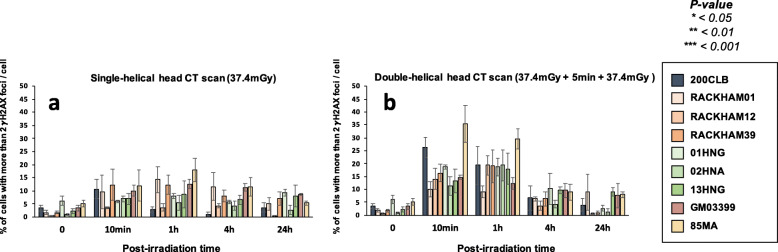
Average number of cells with more than 2 γH2AX foci in fibroblasts after single- and double-helical CT scan exposure. Data shown in Fig. 3 but expressed as the number of cells with more than 2 γH2AX foci after single- (**a**) or double-helical (**b**) CT scan exposure. Each data represents the mean ± SEM of three independent experiments. *CT* Computed tomography, *γH2AX* Phosphorylated forms of the H2AX histone variant, *SEM* Standard error of the mean

In our hands, significant biological effects are observed in cells that show more than two γH2AX foci [16] (Fig. 4). In agreement with the data described above, all the cell lines derived from radio-sensitive/susceptible patients showed more than two γH2AX foci than controls, supporting again a great variety of individual responses to CT scan exposure.

**Fig. 5:**
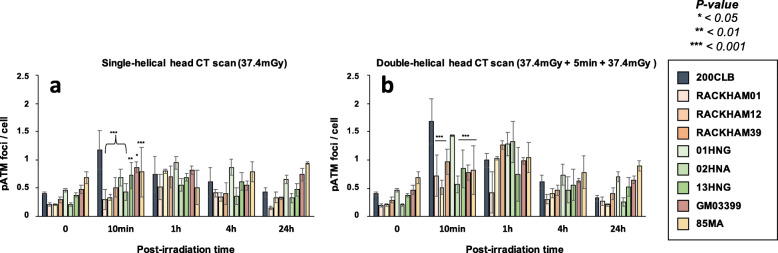
pATM foci in fibroblasts after single- and double-helical CT scan exposure. **a** Kinetics of pATM foci in fibroblasts after a single-helical head CT scan, or (**b**) a double-helical head CT scan at the indicated times after exposure (t0 = non-irradiated). Each data represents the mean ± SEM of 3 independent experiments. The asterisks shown at 10 min post-irradiation times correspond to a statistically significant difference between the cell line and the radioresistant 200CLB controls. *CT* Computed tomography, *γH2AX* Phosphorylated forms of the H2AX histone variant, *pATM* Phosphorylated forms of the ATM protein, *SEM* Standard error of the mean

Since a normal nuclear ATM activity is required for the formation of γH2AX foci, we have also investigated the number of nuclear pATM foci assessed in the same experimental conditions than those described above. Again, accordingly with γH2AX data, the pATM data consolidated our conclusions with regard to the diversity of the responses to both single- and double-helical CT scan conditions (Figs. 5 and S5). The Table 2 summarised the results obtained in the two conditions of CT scan exposure.

### Radiobiological effects of single- and double-helical head CT scans on human astrocytes

The same head CT scan exposure conditions were applied to brain astrocytes. For astrocytes, the average CTDIvol was 27.3 ± 1.99 mGy. The DLP was 441 ± 36 mGy.cm. The average absorbed dose in the phantom was 27.2 ± 2.5 mGy and 55.9 ± 5.72 mGy for the single- and double-helical conditions.

**Fig. 6:**
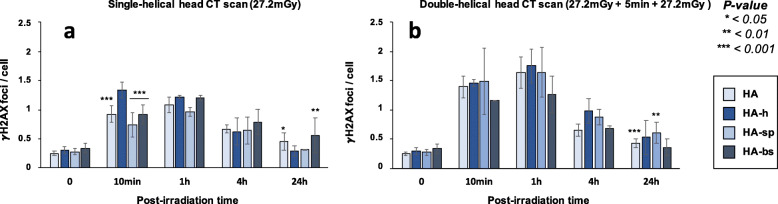
γH2AX foci in astrocytes after single and double-helical CT-scan exposure. **a** Kinetics of γH2AX foci in astrocytes after a single helical head CT scan, or (**b**) a double-helical head CT scan at the indicated times after exposure (t0 = non-irradiated). Each data represents the mean ± SEM foci per cell of 3 independent experiments. The asterisks shown at 10 min post-irradiation times correspond to a statistically significant difference between HA-h and the three other cell lines. For time 24 h, asterisks correspond to a statistically significant difference from the non-irradiated conditions for the same cell line. *CT* Computed tomography, *γH2AX* Phosphorylated forms of the H2AX histone variant molecular, *SEM* Standard error of the mean

No statistical difference was observed between the numbers of spontaneous γH2AX foci assessed in the 4 brain cell lines tested (less than 0.5 spontaneous γH2AX foci was scored per cell). It is noteworthy that the numbers of spontaneous γH2AX foci were similar to that observed with the radioresistant 200CLB control fibroblasts (Figs. 3a and 6a).

Ten minutes after a single-helical CT scan exposure, the hippocampus astrocytes (HA-h) elicited 1.33 ± 0.13 γH2AX foci per cell. This value was slightly higher than the currently reported rate of DSB induced per Gy per human diploid fibroblast (37 ± 4 γH2AX foci per Gy per cell [16]: the expected value would have been 1.01 ± 0.10 γH2AX foci per cell after 27.2 mGy) (Fig. 6a). The three other brain astrocytes cell lines showed similar γH2AX foci data with less than one γH2AX foci per cell, but significantly less than the HA-h astrocytes (*p* < 0.001) (Fig. 6a). Interestingly, while the hippocampus astrocytes elicited a maximal number of γH2AX foci at 10 min post-irradiation higher than the other astrocytes cell lines, the number of γH2AX foci decreased with repair time to reach the lowest number of residual γH2AX foci at 24 h post-irradiation, suggesting both complete DSB recognition and repair processes. All the other astrocytes showed a maximal number of γH2AX foci at 1 h post-irradiation, suggesting a delayed in the RIANS and therefore an impaired DSB recognition. At 24 h post-irradiation, both cortex and brain stem astrocytes elicited a significant number of residual γH2AX foci (HA, *p* = 0.024; HA-bs, *p* = 0.044), suggesting therefore both impaired DSB recognition and DSB repair. Conversely, spinal cord astrocytes (HA-sp) data suggested impaired DSB recognition but normal DSB repair.

After a double-helical CT scan exposure, the number of γH2AX foci assessed 10 min after irradiation significantly increased for all the astrocytes cell lines (*p* < 0.001) but, like for the fibroblast cell lines tested, the assessed value did not correspond to the double of the number of γH2AX foci obtained after a single-helical CT scan (Fig. 6b). Interestingly, in double-helical CT scan exposure, all the astrocytes reached their maximal number of γH2AX foci at 1 h instead of 10 min post-irradiation, suggesting a deficient DSB recognition early after irradiation.

At 24 h after a double-helical CT scan, only the HA and HA-sp cells showed significantly higher number of residual γH2AX foci than in non-irradiated conditions, suggesting an impairment in the DSB repair (Fig. 6b).

**Fig. 7:**
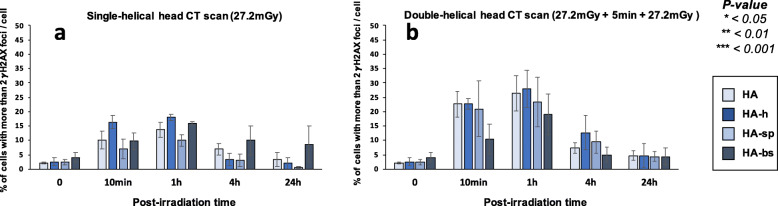
Average number of cells with more than 2 γH2AX foci in astrocytes after single- and double-helical CT scan exposure. Data are shown in Fig. 6 but expressed as the number of cells with more than 2 γH2AX foci after single- (**a**) or double-helical (**b**) CT scan exposure. Each data represents the mean ± SEM of 3 independent experiments. *CT* Computed tomography, *γH2AX* Phosphorylated forms of the H2AX histone variant molecular, *SEM* Standard error of the mean

**Fig. 8:**
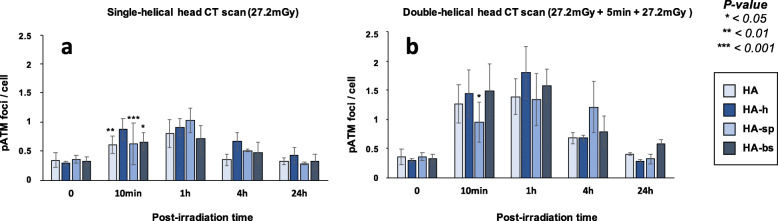
pATM foci in astrocytes after single and double-helical CT-scan exposure. **a** Kinetics of pATM foci in astrocytes after a single helical head CT scan, or (**b**) a double-helical head CT scan at the indicated times after exposure (t0 = non-irradiated). Each data represents the mean ± SEM foci per cell of three independent experiments. Asterisks for time 10 min correspond to a statistically significant difference between HA-h and the other cell lines. *CT* Computed tomography, *γH2AX* Phosphorylated forms of the H2AX histone variant, *pATM* Phosphorylated forms of the ATM protein, *SEM* Standard error of the mean

Finally, the percentages of cells with more than 2 foci γH2AX (Fig. 7) and the numbers of pATM foci (Fig. 8) were assessed in all the conditions tested; the conclusions reached with these two parameters were similar to those suggested by the data described above. Like for fibroblasts, the Table 2 summarised the results obtained and revealed a large diversity of response in astrocytes from the same donor.

## Discussion

For the first time to our knowledge, cutaneous fibroblasts from patients with different levels of radio-sensitivity/susceptibility, and brain astrocytes from the same donor were exposed to single- and double-helical head CT scan sessions. Our findings suggest that individual factors and the nature of tissue, at least, are at the origin of a great diversity of biological response, even at low doses and that the radio-sensitivity/susceptibility may condition the functionality of DSB recognition and repair. Even if the number of cell lines is reduced, the diversity of response should encourage us to investigate further the role of individual factors and tissue-dependence in the final response to CT scan exposure.

In this study, most of the cell lines derived from patients at high risk of cancer and therefore, are supposed to be exposed to CT scan conditions, either for routine CT diagnosis or for tumour imaging before radiotherapy. This is particularly the case of patients with Li-Fraumeni syndrome (*p53*^+/−^ mutations), with heterozygous ataxia telangiectasia (*ATM*^*+/*−^ mutations) and neurofibromatosis type 1 *(NF1*^+/−^ mutations) who represent a non-negligible subset of patients (the corresponding prevalence of those three syndromes is 1/4,000, 1/100, and 1/3,000 on average, respectively). The fibroblasts derived from these syndromes show impaired DSB recognition and/or repair in CT scan exposure conditions but also in radiotherapy exposure conditions [32, 33]. In addition, this study also included three fibroblast cell lines derived from patients showing grade 1-to-4 radiosensitivity after their anticancer radiotherapy (01HNG, 02HNA, 13HNG) [16]. Again, these patients have been submitted to CT scan exposures during their anticancer treatment plan.

Another argument for the necessity of taking into account the individual radio-sensitivity/susceptibility status in the justification of the CT scan exams is provided by the hypersensitivity to low doses phenomenon [34]. This phenomenon shows exacerbated biological effect at a low dose that can correspond to a 5−10 times higher dose [18, 35, 36]. It was shown that this phenomenon preferentially occurs in cells with delayed RIANS. Since this is the case of all the radio-sensitive/radiosusceptible fibroblast cell lines used in this study, it was important to recall the mechanistic model of hypersensitivity to low doses phenomenon: at low dose, less ATM monomers are produced in cytoplasm and less DSB are induced in nucleus. However, if the RIANS is delayed, much less ATM monomers can diffuse to the nucleus. Consequently, few DSB, if any, are recognised and therefore repaired. The unrepaired DSB contribute therefore to the cell lethality but also to RI gene mutations like in an exposure to higher doses [18, 35, 36]. Interestingly, the optimal dose range to observe such the hypersensitivity to low doses phenomenon with the dose-rate applied in head CT scan (100 mGy/min) was found to be [10−50 mGy] [36], which is in very good agreement with head CT scan conditions.

This study involves four human astrocytes cell lines derived from the same donor and representing different regions of the brain. Even if the number of cases is limited, it is noteworthy that our findings revealed, for the first time to our knowledge, different responses to CT scan exposure according to the irradiated part of the brain. The data obtained at low dose reflect the differences observed already at high dose (2 Gy) [37]. Notably, the astrocytes in cortex appeared to be more radiosusceptible (with a high rate of misrepaired DSB) while those in hippocampus showed more radiosensitivity (with a high rate of unrepaired DSB) [37]. Further experiments are needed to establish an actual radiobiological cartography of the brain in order to better define the regions at risk of RI cancers, even after low dose exposure.

All along our investigations, absorbed doses were assessed by a new generation optical scintillating fibre dosimeter developed by the Fibermetrix company (Entzheim, France) and validated in the energy ranges currently used in CT [23]. The dosimetry indicators generally used in the radiobiological studies involving in CT scan, namely CTDI and DLP, show many limitations and are not representative of the dose actually delivered to cells [10, 38]. Hence, our approach allowed us to have more accurate data to provide to the dose-response study. Moreover, given their tightness and their small diameter, these dosimeters permitted to measure reliably the absorbed dose inside the petri dishes on the surface and inside the poly(methyl methacrylate) phantoms (Fig. 2).

Altogether, these data provide a quantitative proof that individual factor should be taken into account in the justification of the CT scan exam. However, additional studies are obviously needed to quantify the risk for a large spectrum genetic statuses and conditions and to better estimate the risks/benefits ratio.

## Supplementary Information

**Additional file 1: Figure S1.** Kinetics of the γH2AX foci in excess in fibroblasts after a single helical head CT scan (a), or a double-helical head CT scan (b) at the indicated post-irradiation times (t0 = non-irradiated). Data result from those shown in Fig. 2 with background subtraction in order to show γH2AX foci in excess effectively due to CT exposure. Error bars indicate SEM. **Figure S2.** Kinetics of pATM foci in excess in fibroblasts (a) after a single helical head CT scan, (b) or a double-helical head CT scan at the indicated post-irradiation times (t0 = non irradiated). Data result from those shown in Fig. 3 with background subtraction in order to show γH2AX foci in excess effectively due to CT exposure. Error bars indicate SEM. **Figure S3.** Kinetics of γH2AX foci in excess in astrocytes (a) after a single helical head CT scan, or (b) a double-helical head CT scan at the indicated post-irradiation times (t0 = non-irradiated). Data result from those shown in Fig. 4 with background subtraction in order to show γH2AX foci in excess effectively due to CT exposure. Error bars indicate SEM. **Figure S4.** Kinetics of the pATM foci in in excess astrocytes (a) after a single helical head CT scan, or (b) a double-helical head CT scan at the indicated post-irradiation times (t0 = non-irradiated). Data result from those shown in Fig. 5 with background subtraction in order to show γH2AX foci in excess effectively due to CT exposure. Error bars indicate SEM. **Figure S5.** Distribution of the number of γH2AX foci per cell over the 300 nuclei scored for the 200CLB and 85MA cell lines at 1h after a single-helical head CT exposure. **Table S1.** Statistical results and *p*-values

## Data Availability

The data presented here are either present in a deposed database (see “Methods” section) or will be made available on reasonable request.

## Abbreviations

ATM: Ataxia telangiectasia mutated gene/protein
CT: Computed tomography
CTDIvol: Volumetric CT dose index
DLP: Dose-length product
DNA: Deoxyribonucleic acid
DSB: DNA double-strand breaks
IR: Ionizing radiation
NF1: Neurofibromatosis type 1 gene/protein/syndrome
pATM: Phosphorylated forms of the ATM protein
RI: Radiation-induced
RIANS: Radiation-induced nucleoshuttling of the ATM protein
SEM: Standard error of the mean
γH2AX: Phosphorylated forms of the H2AX histone variant
