# Supplementary material for: Influence of cellular models and individual factor in the biological response to head CT scan exams
Source: Eur Radiol Exp. 2022 Apr 7;6:17. doi: 10.1186/s41747-022-00269-x (PMC8986906; doi:10.1186/s41747-022-00269-x)
Supplement: Supplementary file 1 — Additional file 1: Figure S1. Kinetics of the γH2AX foci in excess in fibroblasts after a single helical head CT scan (a), or a double-helical head CT scan (b) at the indicated post-irradiation times (t0 = non-irradiated). Data result from those shown in Fig. 2 with background subtraction in order to show γH2AX foci in excess effectively due to CT exposure. Error bars indicate SEM. Figure S2. Kinetics of pATM foci in excess in fibroblasts (a) after a single helical head CT scan, (b) or a double-helical head CT scan at the indicated post-irradiation times (t0 = non irradiated). Data result from those shown in Fig. 3 with background subtraction in order to show γH2AX foci in excess effectively due to CT exposure. Error bars indicate SEM. Figure S3. Kinetics of γH2AX foci in excess in astrocytes (a) after a single helical head CT scan, or (b) a double-helical head CT scan at the indicated post-irradiation times (t0 = non-irradiated). Data result from those shown in Fig. 4 with background subtraction in order to show γH2AX foci in excess effectively due to CT exposure. Error bars indicate SEM. Figure S4. Kinetics of the pATM foci in in excess astrocytes (a) after a single helical head CT scan, or (b) a double-helical head CT scan at the indicated post-irradiation times (t0 = non-irradiated). Data result from those shown in Fig. 5 with background subtraction in order to show γH2AX foci in excess effectively due to CT exposure. Error bars indicate SEM. Figure S5. Distribution of the number of γH2AX foci per cell over the 300 nuclei scored for the 200CLB and 85MA cell lines at 1h after a single-helical head CT exposure. Table S1. Statistical results and p-values [file 41747_2022_269_MOESM1_ESM.docx]

**Influence of cellular models and individual factor in the biological response to head CT scan exams**

**ELECTRONIC SUPPLEMENTARY MATERIAL**

**Figure S1 :**

**
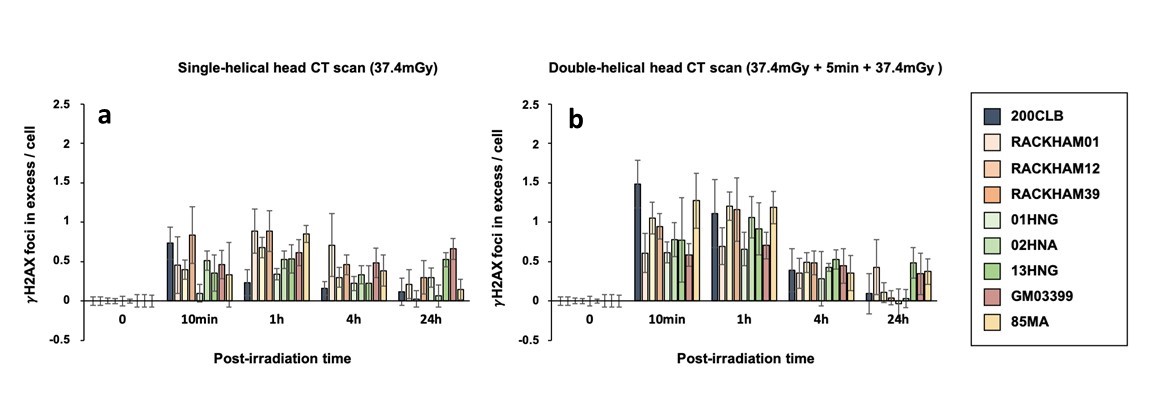
**

**Figure S1: Kinetics of the γH2AX foci in excess in fibroblasts** after a single helical head CT scan **(a)**, or a double-helical head CT scan **(b)** at the indicated post-irradiation times (t0 = non-irradiated). Data result from those shown in Figure 2 with background subtraction in order to show γH2AX foci in excess effectively due to CT exposure. Error bars indicate SEM.

**Figure S2 :**

**
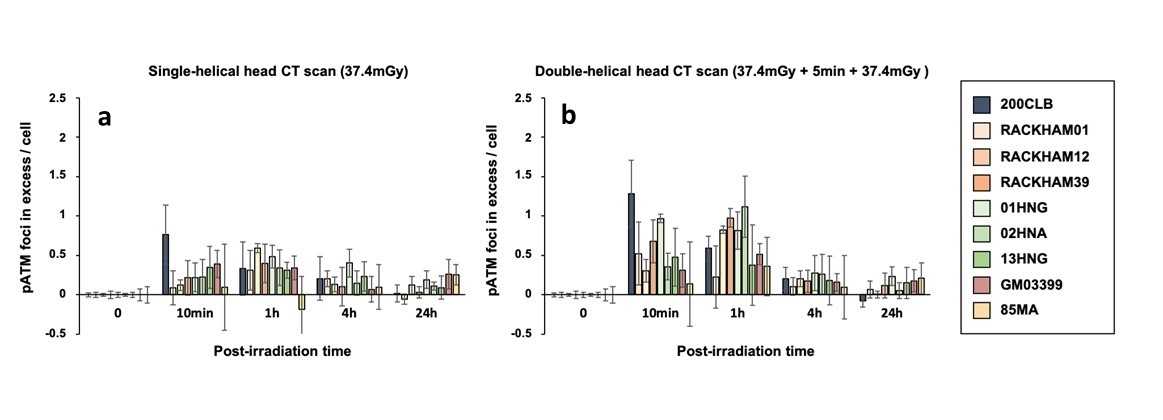
**

**Figure S2:** **Kinetics of pATM foci in excess in fibroblasts** **(a)** after a single helical head CT scan, **(b)** or a double-helical head CT scan at the indicated post-irradiation times (t0 = non irradiated). Data result from those shown in Figure 3 with background subtraction in order to show γH2AX foci in excess effectively due to CT exposure. Error bars indicate SEM.

**Figure S3 :**

**
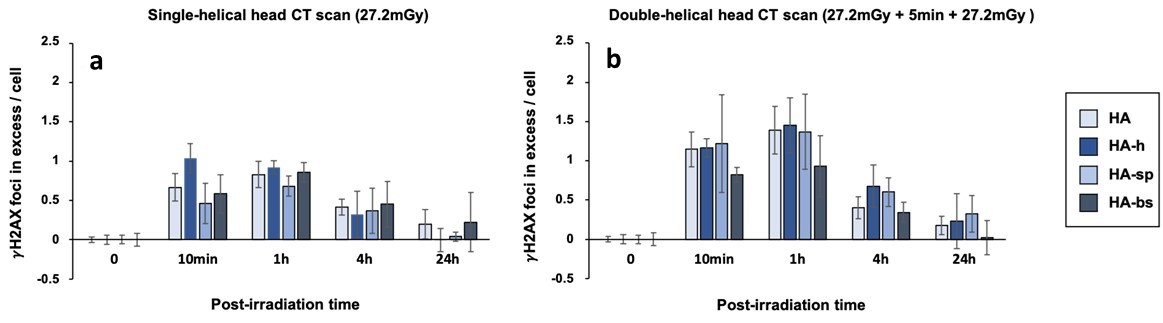
**

**Figure S3: Kinetics of γH2AX foci in excess in astrocytes** **(a)** after a single helical head CT scan, or **(b)** a double-helical head CT scan at the indicated post-irradiation times (t0 = non-irradiated). Data result from those shown in Figure 4 with background subtraction in order to show γH2AX foci in excess effectively due to CT exposure. Error bars indicate SEM.

**Figure S4 :**


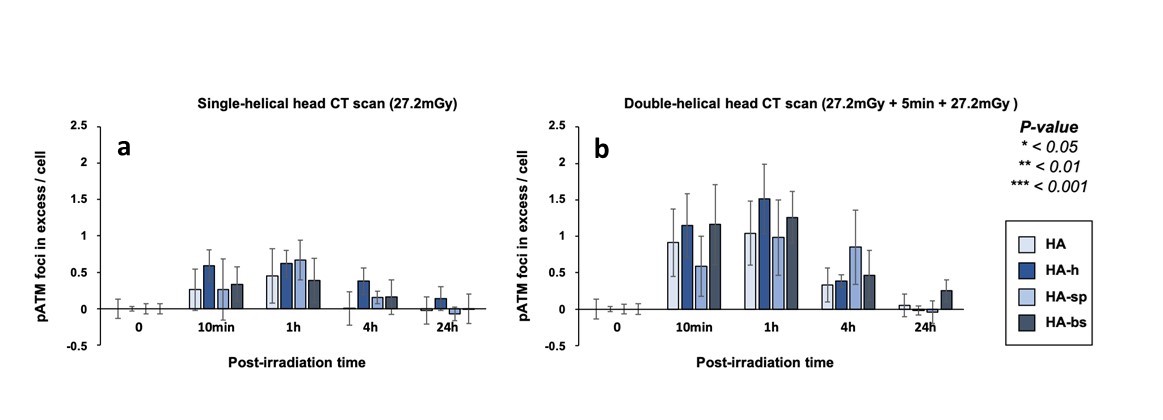


**Figure. S4. Kinetics of the pATM foci in in excess astrocytes** **(a)** after a single helical head CT scan, or **(b)** a double-helical head CT scan at the indicated post-irradiation times (t0 = non-irradiated). Data result from those shown in Figure 5 with background subtraction in order to show γH2AX foci in excess effectively due to CT exposure. Error bars indicate SEM.

**Figure S5 :**


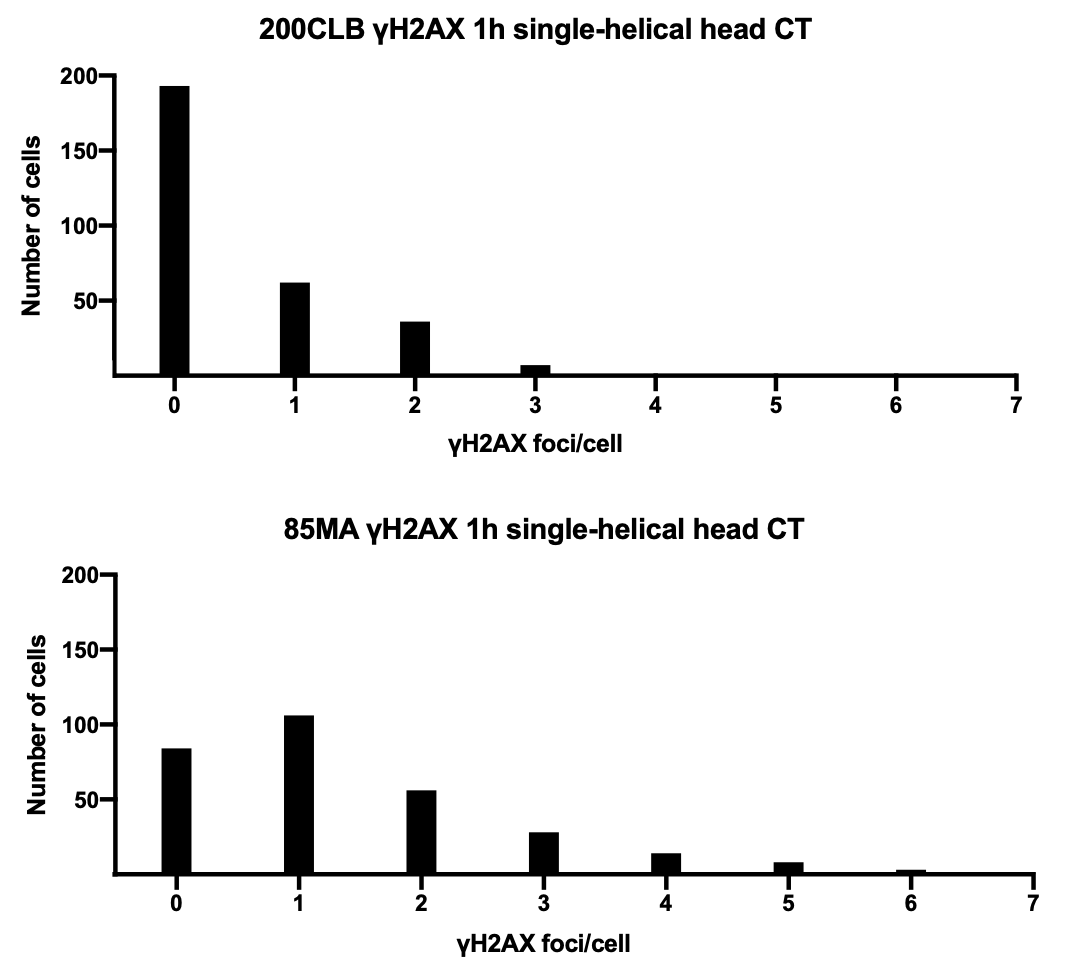


**Figure S5. D**istribution of the number of **γH2AX** foci per cell over the 300 nuclei scored for the 200CLB and 85MA cell lines at 1h after a single-helical head CT exposure.

**Table S1: Statistical results and p-values**

| **Mann-Whitney-Wilcoxon test** | | | | | | | |
| --- | --- | --- | --- | --- | --- | --- | --- |
| **Cell type** | **Cell line** | **Marker** | **Condition** | **Single-Helical** | | **Double-Helical** | |
|  |  |  |  | **Compared with** | **p-values** | **Compared with** | **p-values** |
| **Fibroblasts** | **200CLB** | γH2AX | 24h | 200CLB, non-irradiated | 8.21E-03 | 200CLB, non-irradiated | 2.06E-02 |
|  | **RACKAM01** | γH2AX | Non-irradiated | 200 CLB, non-irradiated | 1.81E-02 | 200 CLB, non-irradiated | 1.81E-02 |
|  |  |  | 10 min | 200 CLB, T= 10min | 9.98E-05 | 200 CLB, T= 10min | 5.33E-11 |
|  |  |  | 1h | 200 CLB, T= 1h | 1.63E-08 | 200 CLB, T= 1h | 2.28E-03 |
|  |  |  | 24h | RACKAM01, non-irradiated | 4.44E-05 | RACKAM01, non-irradiated | 8.36E-12 |
|  |  | pATM | Non-irradiated | 200 CLB, non-irradiated | 1.51E-11 | 200 CLB, non irradiated | 1.51E-11 |
|  |  |  | 10 min | 200 CLB, T= 10min | 1.34E-24 | 200 CLB, T= 10min | 5.09E-11 |
|  | **RACKHAM12** | γH2AX | Non-irradiated | 200 CLB, non-irradiated | 3.03E-02 | 200 CLB, non-irradiated | 3.03E-02 |
|  |  |  | 10 min | 200 CLB, T= 10min | 1.58E-05 | 200 CLB, T= 10min | 1.49E-03 |
|  |  |  | 1h | 200 CLB, T= 1h | 7.16E-07 | 200 CLB, T= 1h | 2.22E-01 |
|  |  |  | 24h | RACKAM12, non-irradiated | 3.46E-01 | RACKAM12, non-irradiated | 3.46E-01 |
|  |  | pATM | Non-irradiated | 200 CLB, non-irradiated | 5.40E-08 | 200 CLB, non irradiated | 1.97E-01 |
|  |  |  | 10 min | 200 CLB, T= 10min | 8.16E-19 | 200 CLB, T= 10min | 2.44E-15 |
|  | **RACKHAM39** | γH2AX | Non-irradiated | 200 CLB, non-irradiated | 6.59E-02 | 200 CLB, non-irradiated | 6.59E-02 |
|  |  |  | 10 min | 200 CLB, T= 10min | 9.59E-01 | 200 CLB, T= 10min | 9.46E-05 |
|  |  |  | 1h | 200 CLB, T= 1h | 6.03E-14 | 200 CLB, T= 1h | 6.08E-01 |
|  |  |  | 24h | RACKAM39, non-irradiated | 4.23E-03 | RACKAM39, non-irradiated | 7.81E-02 |
|  |  | pATM | Non-irradiated | 200 CLB, non-irradiated | 4.38E-04 | 200 CLB, non irradiated | 4.38E-04 |
|  |  |  | 10 min | 200 CLB, T= 10min | 5.16E-12 | 200 CLB, T= 10min | 2.79E-03 |
|  | **01HNG** | γH2AX | Non-irradiated | 200 CLB, non-irradiated | 3.80E-55 | 200 CLB, non-irradiated | 3.80E-55 |
|  |  |  | 10 min | 200 CLB, T= 10min | 1.53E-01 | 200 CLB, T= 10min | 1.86E-03 |
|  |  |  | 1h | 200 CLB, T= 1h | 1.01E-10 | 200 CLB, T= 1h | 6.01E-01 |
|  |  |  | 24h | 01HNG, non-irradiated | 1.53E-05 | 01HNG, non-irradiated | 7.14E-02 |
|  |  | pATM | Non-irradiated | 200 CLB, non irradiated | 1.31E-03 | 200 CLB, non irradiated | 1.31E-03 |
|  |  |  | 10 min | 200 CLB, T= 10min | 7.59E-04 | 200 CLB, T= 10min | 2.66E-01 |
|  | **02HNA** | γH2AX | Non-irradiated | 200 CLB, non-irradiated | 4.57E-01 | 200 CLB, non-irradiated | 4.57E-01 |
|  |  |  | 10 min | 200 CLB, T= 10min | 5.06E-03 | 200 CLB, T= 10min | 2.54E-07 |
|  |  |  | 1h | 200 CLB, T= 1h | 6.57E-03 | 200 CLB, T= 1h | 9.73E-01 |
|  |  |  | 24h | 02HNA, non-irradiated | 6.93E-01 | 02HNA, non-irradiated | 8.97E-01 |
|  |  | pATM | Non-irradiated | 200 CLB, non-irradiated | 2.64E-10 | 200 CLB, non irradiated | 2.64E-10 |
|  |  |  | 10 min | 200 CLB, T= 10min | 1.58E-14 | 200 CLB, T= 10min | 1.28E-13 |
|  | **13HNG** | γH2AX | Non-irradiated | 200 CLB, non-irradiated | 8.00E-09 | 200 CLB, non-irradiated | 8.00E-09 |
|  |  |  | 10 min | 200 CLB, T= 10min | 1.98E-02 | 200 CLB, T= 10min | 1.24E-08 |
|  |  |  | 1h | 200 CLB, T= 1h | 6.94E-05 | 200 CLB, T= 1h | 6.77E-01 |
|  |  |  | 24h | 13HNG, non-irradiated | 1.16E-09 | 13HNG, non-irradiated | 8.92E-10 |
|  |  | pATM | Non-irradiated | 13HNG, non irradiated | 8.87E-01 | 13HNG, non irradiated | 8.87E-01 |
|  |  |  | 10 min | 200 CLB, T= 10min | 1.15E-03 | 200 CLB, T= 10min | 3.54E-06 |
|  | **GM03399** | γH2AX | Non-irradiated | 200 CLB, non-irradiated | 8.12E-25 | 200 CLB, non-irradiated | 8.12E-25 |
|  |  |  | 10 min | 200 CLB, T= 10min | 5.22E-01 | 200 CLB, T= 10min | 2.17E-05 |
|  |  |  | 1h | 200 CLB, T= 1h | 1.62E-13 | 200 CLB, T= 1h | 9.37E-01 |
|  |  |  | 24h | GM03399, non-irradiated | 4.37E-11 | GM03399, non-irradiated | 8.01E-08 |
|  |  | pATM | Non-irradiated | 200 CLB, non irradiated | 1.29E-03 | 200 CLB, non irradiated | 1.29E-03 |
|  |  |  | 10 min | 200 CLB, T= 10min | 4.15E-02 | 200 CLB, T= 10min | 4.43E-06 |
|  | **85MA** | γH2AX | Non-irradiated | 200 CLB, non-irradiated | 2.57E-48 | 200 CLB, non-irradiated | 2.57E-48 |
|  |  |  | 10 min | 200 CLB, T= 10min | 5.39E-01 | 200 CLB, T= 10min | 1.19E-02 |
|  |  |  | 1h | 200 CLB, T= 1h | 6.27E-20 | 200 CLB, T= 1h | 6.87E-06 |
|  |  |  | 24h | 85MA, non-irradiated | 1.54E-02 | 85MA, non-irradiated | 1.87E-11 |
|  |  | pATM | Non-irradiated | 200 CLB, non irradiated | 1.51E-16 | 200 CLB, non irradiated | 1.51E-16 |
|  |  |  | 10 min | 200 CLB, T= 10min | 8.17E-04 | 200 CLB, T= 10min | 4.03E-08 |
| **Astrocytes** | **HA-h** | γH2AX | 24h | HA-h, non-irradiated | 5.27E-01 | HA-h, non-irradiated | 2.81E-01 |
|  | **HA** | γH2AX | Non-irradiated | HA-h, non-irradiated | 6.53E-02 | HA-h, non-irradiated | 6.53E-02 |
|  |  |  | 10 min | HA-h, T= 10min | 4.48E-04 | HA-h, T= 10min | 1.29E-01 |
|  |  |  | 1h | HA-h, T= 1h | 4.42E-01 | HA-h, T= 1h | 1.49E-01 |
|  |  |  | 24h | HA, non-irradiated | 5.00E-02 | HA, non-irradiated | 8.41E-04 |
|  |  | pATM | Non-irradiated | HA-h, non irradiated | 1.34E-01 | HA-h, non irradiated | 1.34E-01 |
|  |  |  | 10 min | HA-h, T= 10min | 5.71E-03 | HA-h, T= 10min | 2.38E-02 |
|  | **HA-sp** | γH2AX | Non-irradiated | HA-h, non-irradiated | 2.16E-01 | HA-h, non-irradiated | 2.16E-01 |
|  |  |  | 10 min | HA-h, T= 10min | 2.11E-08 | HA-h, T= 10min | 1.32E-01 |
|  |  |  | 1h | HA-h, T= 1h | 6.24E-02 | HA-h, T= 1h | 1.12E-01 |
|  |  |  | 24h | HA-sp, non-irradiated | 2.37E-02 | HA-sp, non-irradiated | 2.32E-03 |
|  |  | pATM | Non-irradiated | HA-h, non irradiated | 2.07E-01 | HA-h, non irradiated | 2.07E-01 |
|  |  |  | 10 min | HA-h, T= 10min | 2.88E-05 | HA-h, T= 10min | 2.94E-05 |
|  | **HA-bs** | γH2AX | Non-irradiated | HA-h, non-irradiated | 5.41E-01 | HA-h, non-irradiated | 5.41E-01 |
|  |  |  | 10 min | HA-h, T= 10min | 4.33E-04 | HA-h, T= 10min | 6.46E-03 |
|  |  |  | 1h | HA-h, T= 1h | 6.72E-01 | HA-h, T= 1h | 8.30E-05 |
|  |  |  | 24h | HA-bs, non-irradiated | 4.10E-03 | HA-bs, non-irradiated | 3.77E-01 |
|  |  | pATM | Non-irradiated | HA-h, non irradiated | 4.62E-01 | HA-h, non irradiated | 4.62E-01 |
|  |  |  | 10 min | HA-h, T= 10min | 3.38E-02 | HA-h, T= 10min | 6.93E-01 |
| **Kruskal-Wallis test** | | | | | | | |
| **Cell type** | | **Marker** | **Condition** | **Single-Helical** | | **Double-Helical** | |
|  |  |  |  | **p-values** | | **p-values** | |
| Fibroblasts | | γH2AX | 10 min | 2.29E-10 | | 6.85E-27 | |
|  |  |  | 1h | 1.87E-21 | | 6.76E-12 | |
|  |  |  | 24h | 8.52E-59 | | 4.48E-62 | |
|  |  | pATM | 10 min | 3.87E-21 | | 1.70E-30 | |
| Astrocytes | | γH2AX | 10 min | 2.50E-07 | | 6.19E-02 | |
|  |  |  | 1h | 2.86E-01 | | 1.30E-03 | |
|  |  |  | 24h | 7.27E-02 | | 4.87E-01 | |
|  |  | pATM | 10 min | 2.88E-04 | | 8.81E-05 | |
